# The non-adiabatic nanoreactor: towards the automated discovery of photochemistry†

**DOI:** 10.1039/d1sc00775k

**Published:** 2021-04-29

**Authors:** Elisa Pieri, Dean Lahana, Alexander M. Chang, Cody R. Aldaz, Keiran C. Thompson, Todd J. Martínez

**Affiliations:** Department of Chemistry, The PULSE Institute, Stanford University Stanford CA 94305 USA toddjmartinez@gmail.com; SLAC National Accelerator Laboratory 2575 Sand Hill Road Menlo Park CA 94025 USA

## Abstract

The *ab initio* nanoreactor has previously been introduced to automate reaction discovery for ground state chemistry. In this work, we present the nonadiabatic nanoreactor, an analogous framework for excited state reaction discovery. We automate the study of nonadiabatic decay mechanisms of molecules by probing the intersection seam between adiabatic electronic states with hyper-real metadynamics, sampling the branching plane for relevant conical intersections, and performing seam-constrained path searches. We illustrate the effectiveness of the nonadiabatic nanoreactor by applying it to benzene, a molecule with rich photochemistry and a wide array of photochemical products. Our study confirms the existence of several types of S_0_/S_1_ and S_1_/S_2_ conical intersections which mediate access to a variety of ground state stationary points. We elucidate the connections between conical intersection energy/topography and the resulting photoproduct distribution, which changes smoothly along seam space segments. The exploration is performed with minimal user input, and the protocol requires no previous knowledge of the photochemical behavior of a target molecule. We demonstrate that the nonadiabatic nanoreactor is a valuable tool for the automated exploration of photochemical reactions and their mechanisms.

## Abstract 2

The nonadiabatic nanoreactor is a tool for automated photochemical reaction discovery that extensively explores intersection seams and links conical intersections to photoproduct distributions.

## Graphical abstract

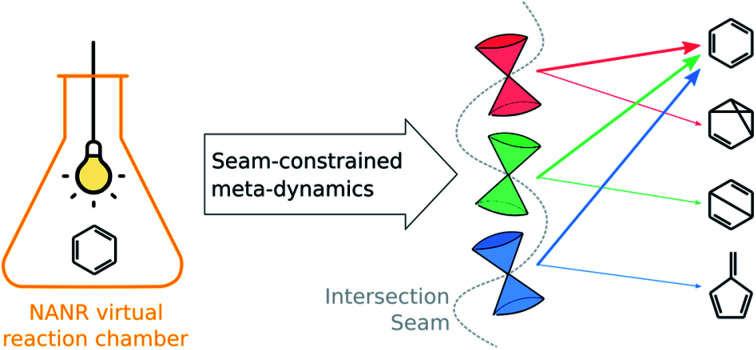

## Introduction

The potential of light to promote chemical reactions was first recognized at the beginning of the 20^th^ century, when Ciamician urged industry to investigate “the photochemistry of the future”.^1^ Indeed, light-driven syntheses can allow for milder reaction conditions, shorter synthetic routes and avoidance of protection/deprotection for functional groups.^2^ In some cases, photoreactions can produce a low number of side products, resulting in enhanced atom economy. The formation of highly reactive species in a controlled manner has led some to tout the photon as “the greenest, traceless reagent in organic synthesis”.^3^ Furthermore, light has become increasingly popular as a synthetic tool through systematic multidimensional reaction screening:^4^ powerful, high-throughput experimental setups are exploited to experiment with different wavelengths, solvents, sensitizers, and irradiation time. This allows chemists to venture into unexplored territories of both chemical and reaction space, and to discover new synthetic routes. In order to achieve the future of photochemistry that Ciamician envisioned, however, the rational design and discovery of novel photosynthetic routes must be informed by an accurate understanding of the underlying theoretical chemistry. Furthermore, the promise of theoretical chemistry to uncover photochemical mechanisms and product distributions at scale (for example as an *in silico* analogy to the photochemical farms of Ciamician) is highly appealing, especially if used in conjunction with well-thought-out experiments through a feedback mechanism.

Computational chemistry currently provides numerous automated tools to guide thermochemical reaction discovery.^5–8^ One such example is the *ab initio* nanoreactor,^9–13^ where accelerated molecular dynamics is employed to prompt the discovery of new reactions between molecules in a virtual reaction chamber, without the need for pre-existing knowledge of the system under investigation. Discovered reactions are then refined at a higher level of theory to produce a kinetic model based on the generated network of reactions and relevant thermodynamic quantities. The nanoreactor and related methods^7,8,14–16^ are strongly rooted in the use of molecular dynamics simulations to discover new reactions. Alternate approaches that rely on chemical heuristics have also been explored.^6,17–20^

While the aforementioned tools are capable of predicting the chemical outcome in a reaction vessel under various thermal conditions, they cannot be directly applied to photochemical reactions. In light-driven processes, reactions take place far from equilibrium and the usual transition state theory (TST) underlying ground state methods is not obviously applicable. Although some have drawn an analogy between minimal energy conical intersections (MECIs) for excited state reactions and transition states for ground state reactions, this analogy is imperfect because the starting point in an excited state reaction (*e.g.* the Franck–Condon (FC) region) is usually energetically far above the relevant conical intersection (CI). Thus, one expects that many points along the intersection seam (roughly analogous to the dividing surface in TST) will be accessed and the MECI may not be accessed at all.^21^ Many notable efforts have been devoted to the development of a nonadiabatic transition state theory;^22–26^ however, the theory remains incomplete.

Despite the complexity of intersecting potential energy surfaces (PESs), nonadiabatic processes have attracted many theoretical chemists. For example, Aldaz *et al.* used combinatorically generated reaction coordinates to search for MECIs and then followed the ground-state down to photoproducts.^27^ Others have developed tools for the systematic exploration of the intersection seam using modified potential energy surfaces and molecular dynamics. For example, Maeda *et al.*^28^ combined the seam model function with multicomponent artificial force induced reaction (SMF/MC-AFIR) to automatically identify and characterize large numbers of available CIs. This method removes the arbitrariness typically introduced by choosing an initial geometry guess for an MECI search; therefore, one can start the search from the FC point and systematically explore configurational space for CIs. Similarly, Lindner *et al.* recently developed the metaFALCON^29^ approach, using multistate metadynamics to systematically scan the intersection space and find MECIs. In this work, we also take the metadynamics approach, and by leveraging the powerful toolsets within the nanoreactor we show how this can be made into a useful toolset for photochemical discovery.

Further work has been dedicated to better understand the impact of the CI topography^30^ on the transition probability. Some studies found evidence of transition efficiency's sensitivity to topography, where peaked conical intersections have been observed transferring population more efficiently than sloped ones.^31^ In some cases, sloped conical intersections are found to be good facilitators for lower state to upper state transitions (referred to as “up-funneling” or “diabatic trapping”) due to their low efficiency in directing away the quenched wavepacket.^32–35^ Other studies have emphasized the importance of dynamical effects and the direction from which the molecule approaches the CI.^36^ Although it is still unclear whether any general statements can be made about the dominance of any of these effects (topography, approach direction, velocity) on the photochemical outcome, it is certainly clear that they are important.

Despite these contributions, modeling a photoreaction from photon absorption to photoproduct formation is not a trivial task. Currently there is only one comprehensive method capable of fully modeling the evolution of a photoexcited molecule from the FC point to its photoproduct(s): quantum dynamics, to which *ab initio* nonadiabatic molecular dynamics (NA-MD) is a good approximation.^37,38^ Independent of the various implementations of NA-MD, finding robust quantum chemical theory that is able to correctly describe the excited state, especially in regions of strong nonadiabatic coupling such as the intersection seam, remains a significant hurdle. Additionally, these methods are typically computationally demanding, and non-negligible work is required prior to the dynamics calculations to validate the chosen computational method. Moreover, capturing rare events, which translates to being able to observe the formation of low quantum yield photoproducts, requires the computation of a large number of NA-MD trajectories with different initial conditions (and even then, it is difficult to guarantee complete sampling).

In this work, taking inspiration from the thermal *ab initio* nanoreactor, we present the nonadiabatic nanoreactor (NANR), a tool for the automatic exploration of photochemistry that aims at modeling photoreactions with no prior chemical knowledge. Using benzene as an example application, we show how enhanced sampling techniques and path refinement yield qualitative information on the outcome of photoexcitation, while avoiding nonadiabatic dynamics altogether. We also explore the relationship between conical intersection topography and product distributions.

### Theory and methods

The NANR workflow is inspired by the ground state *ab initio* nanoreactor,^9^ as it features a discovery phase and a refinement phase. The molecule under investigation is confined in a virtual reaction chamber, and we simulate photoexcitation to different electronic states and ensuing photochemical reactions. The same virtual reaction chamber concept can be used to study bimolecular photoreactions and/or explicit solvation, in analogy with the ground state *ab initio* nanoreactor setup.

In the discovery phase, the goal is to extensively explore the intersection seam between two electronic states to identify and characterize important classes of conical intersections. A key aim of the NANR is to capture rare photochemical events in order to be able to recover all possible outcomes of a photochemical reaction. Therefore, it is important to be able to explore all important regions of the seam space, including higher energy regions or regions separated by high energy barriers which might only be reached rarely. In this phase, we seek to understand which photoproducts are accessible from a given conical intersection. In the refinement phase, path calculation techniques are used to gain information about the topography of the potential energy surface(s) and the intersection seam. This provides a way to generate qualitative hypotheses on the accessibility of certain regions of the configurational space, while avoiding the calculation of nonadiabatic dynamics.

**Fig. 1:**
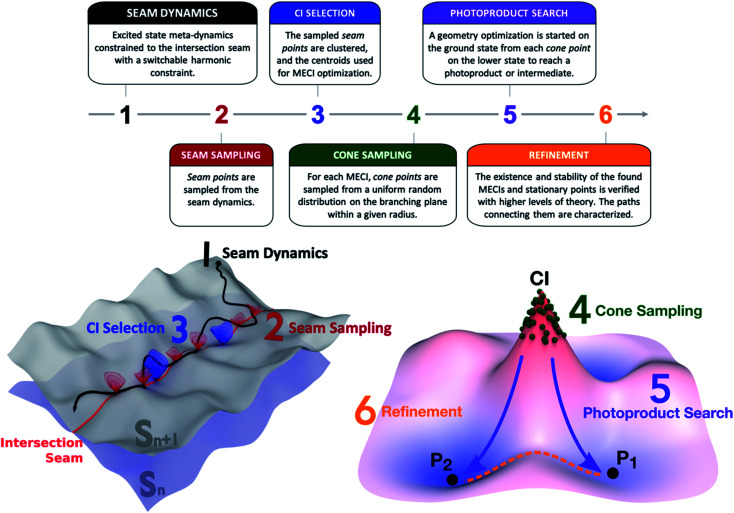
NANR workflow: the first five steps constitute the discovery phase; the last step is a refinement phase. Steps 1 to 3 deal with the exploration of the intersection seam between two potential energy surfaces and the selection of representative CIs. Steps 4 to 5 focus the attention on the lower electronic state, by investigating the photoproduct distribution of each relevant CI, and the minimum energy paths connecting the important points of the PESs.

The NANR workflow consists of six steps (see Fig. 1). In the first step (Seam Dynamics), metadynamics^39,40^ is used to accelerate the discovery phase by propagating a trajectory starting from the ground state equilibrium geometry on the excited state with no initial velocity and using a Bussi–Parrinello thermostat.^41^ Metadynamics can informally be described as “filling the free energy wells with computational sand”.^42^ The system is described by a low number of collective variables; while the molecule evolves on the energy surface, a Gaussian-shaped biasing potential is added periodically at the molecule's location.^43^ The technique enables the molecule to overcome barriers between energy wells, therefore enhancing conformational sampling, and can provide information on free energy profiles and other state functions. Following Grimme,^44^ we use the root-mean-square deviation (RMSD) between optimally aligned frames (calculated using a quaternion algorithm^45^) as the collective variable in our metadynamics implementation. A constraint is used to bind the molecule to the intersection seam between the two desired states. We use a similar method to that employed by Mori *et al.* to constrain nudged elastic band (NEB) searches to the intersection seam.^46^ In our case, the constraint is linear when the energy difference between the two states is above a user-defined threshold and harmonic when below this threshold, as described in detail in the ESI.† These aforementioned features contribute to the exploratory nature of this step: the metadynamics aspect, together with the seam-constraint, allows us to thoroughly inspect a large portion of the seam space lying energetically below the FC point. As usual, it is difficult to prove that one has obtained complete sampling with finite computational resources, but the metadynamics procedure has proved quite effective in practice. The Seam Dynamics step was performed on benzene at 300 K using a complete active space configuration interaction method built on floating occupation molecular orbitals (FOMO-CASCI)^47–50^ with six electrons in five active orbitals and the 6-31G basis set, *i.e.* FOMO-CASCI(6/5)/6-31G. The dynamics starts on S_1_ at the FC geometry to explore the S_0_/S_1_ intersection space. A plot of the explored conformational wells and more detail on this step can be found in the ESI (Fig. S1 and Section SI-I†).

The second step (Seam Sampling, Fig. 1) involves the collection of all the points along the intersection seam where the energy gap between the two electronic states is lower than 0.1 eV. This process potentially yields a large number of molecular structures, which are pruned in the third step, CI selection. The CI selection phase is crucial, as we have observed that very small geometric differences can greatly impact the topography of the conical intersection. For these reasons, we aim to identify the best set of representative structures in our large initial set, while still keeping the number of subsequent calculations manageable. In the current workflow, the structures sampled from the seam dynamics are optimally aligned using rotation and label permutations to treat the problem posed by benzene's symmetry. Once their cartesian RMSD with respect to a refence structure is minimized, the structures are clustered together based on their Euclidean distance using the K-means algorithm. The cluster analysis uses the Euclidean distance between the geometries as the distance function; the elbow method^51^ is used to decide the optimal number of clusters to be used for dividing the geometries (which we found to be six for the cases discussed in this paper). The centroids of the clusters are used as starting guesses for a MECI search using the Lagrange-Newton method.^52^ It is important to note that MECIs are often not directly involved in the nonadiabatic decay pathway since many regions of the intersection seam are usually easily accessed from the FC point. This means other CIs are often preferred for population transfer over the MECIs, which might not even be reached during a photochemical process. However, MECIs offer the great advantage of being unique and readily identifiable: an extended region of the seam can be characterized by its local minimum, whose geometry is often representative of the surrounding basin.^21^ Therefore, our protocol collects all potentially accessible CI types and represents them with their closest MECI. Other dimensionality reduction techniques can be considered in the future to select representative CIs.

The fourth step in the NANR workflow is Cone Sampling, the systematic nature of which is important for a robust discovery, including rare events. For each of the MECIs found in the previous step, the orthonormalized^53^**g** and **h** vectors (*i.e.* the two coordinates, representing the gradient of the energy difference and the product of the energy difference and nonadiabatic coupling vector,^53^ along which the energy degeneracy is lifted linearly at a CI) are used to build the branching plane around the conical intersection. Cone points are selected on the branching plane according to a random sampling scheme:c⃑

1 *r⃑* = *r⃑*_0_ + *l* cos(*α*) *g⃑* + *l* sin(*α*)*h⃑*

with

2 *l* = *cR*

3 *α* = 2*b*π

where *r⃑* and *r⃑*_0_ are the sampled and CI geometries, *b* and *c* are chosen from a uniform random distribution on the interval (0,1), and the radius *R* is decided by the user. This step provides a systematic mapping of the branching space. For each of the identified S_0_/S_1_ MECIs, during the Cone Sampling we select 500 cone points around the MECI, choosing *R* such that the energy gap between the two electronic states ranges between 0.0 and 0.5 eV (*R* = 0.075).

In the next phase, the Photoproduct Search (step 5, Fig. 1), each of the sampled cone geometries is optimized on the lower state to reach stationary points on the potential energy surface. These optimizations minimize the potential energy by following the gradient and do not include dynamic effects. The focus of this step is discovering which stationary points are reachable from a given CI rather than providing accurate quantitative information. It is likely that adding dynamics calculations (either with no initial velocity or with initial velocities sampled using a second order description of the seam^54,55^) would change the photoproduct ratio, since velocity helps to overcome low and medium barriers and typically favors lower energy local minima. Similarly, the inclusion of nuclear quantum effects could potentially impact the results of this step. Finally, one could use the ground state nanoreactor framework (potentially with metadynamics) in order to more extensively explore the fate of photoproducts. These aspects will be explored in further work.

Finally, the workflow features a Refinement step. While the previous five steps are performed using a relatively inexpensive level of theory, such as FOMO-CASCI, during Refinement, the energy, stability, and geometry of identified MECIs and stationary points are verified with more accurate methods, such as XMS-CASPT2. This step is necessary in order to establish the quality of the potential energy surfaces at the lower level of theory used for sampling and to validate the results provided in the previous steps. In Refinement, minimum energy paths connecting pairs of MECIs on the seam space and stationary points on the ground state are computed using the growing string method (GSM).^27,56^ This allows us to identify barriers within the seam and make hypotheses on the accessibility of a given CI type.

The protocol described above represents the workflow followed to explore the S_0_/S_1_ intersection space and find photoproducts. When the exploration starts from a higher electronic state S_*n*_ (where *n* > 1), a few minor modifications are required: the Seam Dynamics step is calculated on S_*n*_ with a constraint binding the molecule to the S_*n*_/S_*n*−1_ intersection seam. The Seam Sampling, CI selection and Cone Sampling proceed as described above, with a smaller number of sampled cone points. Then, rather than continuing with the Photoproduct Search, the protocol rewinds to the Seam Dynamics step, where a trajectory is calculated for each of the sampled cone points on S_*n*−1_, to explore the S_*n*−1_/S_*n*−2_ intersection space. This subset of steps is repeated in a cascade mechanism until reaching S_1_, where the cycle terminates, leading to the Photoproduct Search and Refinement phases. In our study, when the discovery was started from the second singlet excited state, the seam-constrained metadynamics trajectory was propagated on S_2_ at 300 K from the FC point; 10 cone points with an energy gap between 0.1 and 0.5 eV were sampled around each identified S_1_/S_2_ MECI and used as seeds for the subsequent 2 ps long S_0_/S_1_ seam dynamics calculations.

**Fig. 2:**
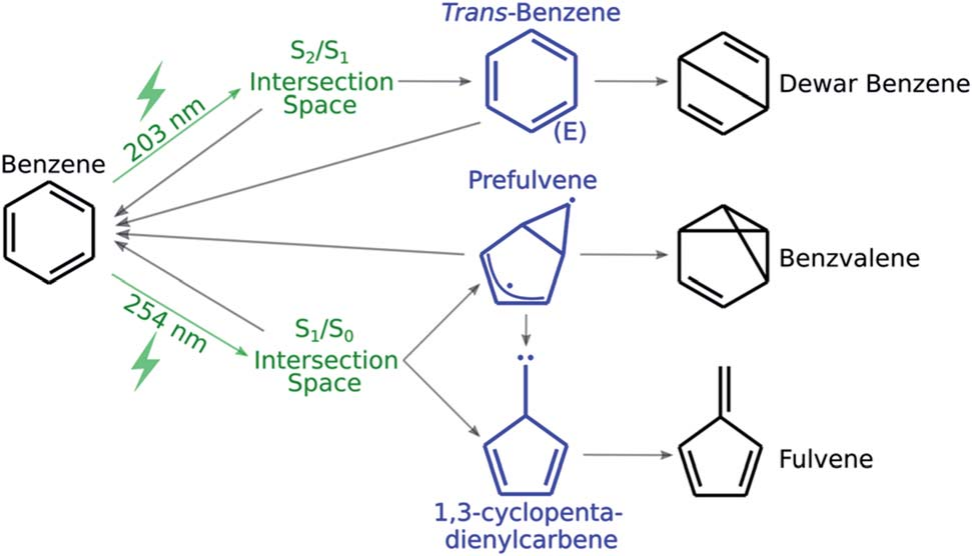
A schematic representation of benzene's known photochemistry. Photoexcitations are depicted in green, transient species in blue and stable photoproducts in black.

As a first application of the NANR workflow, we explore the photochemistry of benzene. Despite being a small molecule, benzene offers a rich photochemical activity (see Fig. 2): depending on the wavelength of the light used for the excitation, several minor photoproducts can be formed as a result of the four quasi-unpaired electrons that characterize low energy wells of the S_0_/S_1_ seam space.^57^ These characteristics make benzene an ideal candidate, regarding size and photoproduct variety, for our first validation.

All the electronic structure calculations used in this work are performed using the TeraChem^58–60^ software package and pyGSM^27^ is used for optimization of minimum energy paths. The use of GPU-accelerated code makes the global computational effort very affordable. Overall, the whole workflow is capable of providing qualitative results in a fraction of the time required by nonadiabatic dynamics simulations with the same level of theory. In this first demonstration of the NANR, the system is studied in gas phase. Extensions to include implicit or explicit solvation are in progress.

## Results and discussion

### Seam dynamics, seam sampling and CI selection results

The photoactivity of benzene has been experimentally monitored upon photoexcitation with two UV wavelengths (254 nm and 203 nm), revealing different photoproduct distributions.^61^ The former wavelength promotes the molecule to the first singlet excited state, S_1_ (^1^B_2u_), the latter promotes to the second singlet excited state, S_2_ (^1^B_1u_). For this reason, we chose to investigate both the S_1_/S_2_ and S_0_/S_1_ intersection seams with the nonadiabatic nanoreactor.

Benzene has served as a prototypical system for the study of conical intersections;^62^ its lowest energy MECI has been reported several times in literature,^29,62–65^ characterized by a half-boat-like geometry with equatorial ring puckering. Other tools have been applied to systematically scan the S_0_/S_1_ seam space,^63^ or to discover high energy CI types by applying symmetry operations.^65,66^ A common feature of the MECIs reported in these works is the presence of symmetry (often C_s_) in the geometrical structures.

**Fig. 3:**
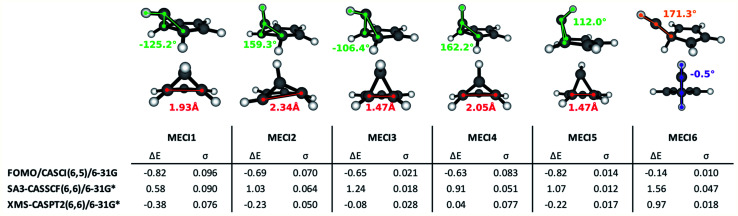
Geometric structures and properties, energy values and slopeness of the S_0_/S_1_ identified MECIs. The pyramidalization dihedral value of the out-of-plane carbon atom is indicated in green; the distance between the bridge carbon atoms is indicated in red, in Angstroms; other interesting dihedrals and angles are pictured in violet and orange. Δ*E* is the energy difference (in eV) between the S_1_ energy at the FC point and the energy of the degenerate electronic states at the CI. *σ* is the slopeness parameter.

The execution of steps 1 and 2 of the nonadiabatic nanoreactor – Seam Dynamics and Seam Sampling – for benzene as described in the Methods section yielded ∼21 000 seam geometries. Clustering and subsequent MECI searches from the cluster centroids, as per step 3, CI selection, identified 6 distinct MECIs (Fig. 3); in all of them, the planarity of the benzene ring is lost, as previously found in the literature.

The lowest energy and best known benzene MECI^29,62,64^ is here labeled MECI1; the geometry retains C_s_ symmetry. MECI2, previously reported by Lindner *et al.*,^29^ lies slightly higher in energy (∼0.1 eV) with respect to MECI1, and features symmetry breaking. MECI3 is similar geometrically to MECI1 and energetically to MECI2; in this molecular geometry, the carbon–carbon distance for the pair of atoms at the base of the three-atom ring is reduced to 1.47 Å, which is comparable to the classic carbon–carbon single bond length. To our knowledge, this MECI has not been described before. MECI4 features C_s_ symmetry, but is ∼0.2 eV higher in energy than MECI1, due to the pyramidalization inversion of the out-of-plane carbon atom. This MECI has been previously reported by several studies.^29,63,65^ MECI5, whose energy is comparable to MECI1, is geometrically very similar to MECI3, but features once again a pyramidalization inversion of the out-of-plane carbon atom. As with MECI3, we are not aware of previous studies describing this MECI. Finally, MECI6 is characterized by a carbene-like structure with C_s_ symmetry; this MECI has a higher energy compared to the others (nearly 0.7 eV higher than MECI1), and was previously discovered by the MetaFALCON package.^29^

Because we limit exploration to MECIs that are energetically accessible from the FC point, we did not find the high-energy MECIs previously reported in literature by using symmetry operations.^65^ In our study, all six of the MECIs lay below the FC point S_1_ energy at the FOMO-CASCI level of theory, which could indicate good accessibility. However, in order to verify their effective accessibility, minimum energy paths should be calculated between the FC point and each MECI in order to estimate the size of any barriers.

Each of the FOMO-CASCI MECIs was then re-optimized with SA3-CASSCF(6,6)/6-31G* and XMS-CASPT2(6,6)/6-31G*, showing no significant structural change. Curiously, CASSCF places all of these MECIs above the FC point S_1_ energy, while XMS-CASPT2 energy trends are in agreement with the FOMO-CASCI ones (see Fig. 3). This discrepancy is due to the (6,6) “intuitive” active space used for the CASSCF calculations, which is known to overestimate the S_2_-S_1_ energy gap at the FC point.^67^ Smaller active spaces, such as the (6,5) space used in our FOMO-CASCI exploration step for benzene, have been shown to provide a more balanced treatment of the lowest two singlet excited states in benzene^67^ and polyenes.^68^ The good agreement between FOMO-CASCI and XMS-CASPT2 *versus* CASSCF confirm that FOMO-CASCI(6,5)/6-31G is an adequate level of theory to describe benzene's photochemistry, and should be preferred over CASSCF(6,6)/6-31G*.

Another characteristic that is monitored in the workflow is the local CI topography. We use a “slopeness” parameter, as introduced by Yarkony.^53^ This can be calculated through the overall slope:4
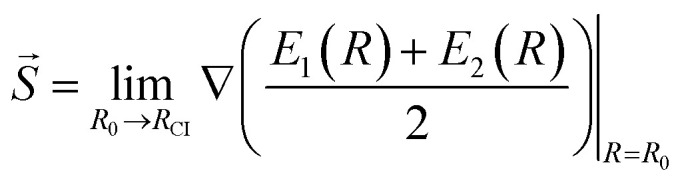
where *E*_1_ and *E*_2_ are the two degenerate electronic states and *R*_CI_ is the CI geometry. The length *σ* of the projection of *S→* on the branching plane quantifies the sloped character of CI: for an ideal peaked intersection, *σ* has a value of zero, while it is larger as the topography becomes more and more sloped. According to the tested levels of theory, the six MECIs identified in this work have a mostly sloped topography at the FOMO-CASCI level of theory, with MECI1 being the most sloped one and MECI6 being the most peaked one. Both the CASSCF and XMS-CASPT2 results are generally in line with the FOMO-CASCI ones, with the exception of the CASSCF prediction on MECI6.

**Fig. 4:**
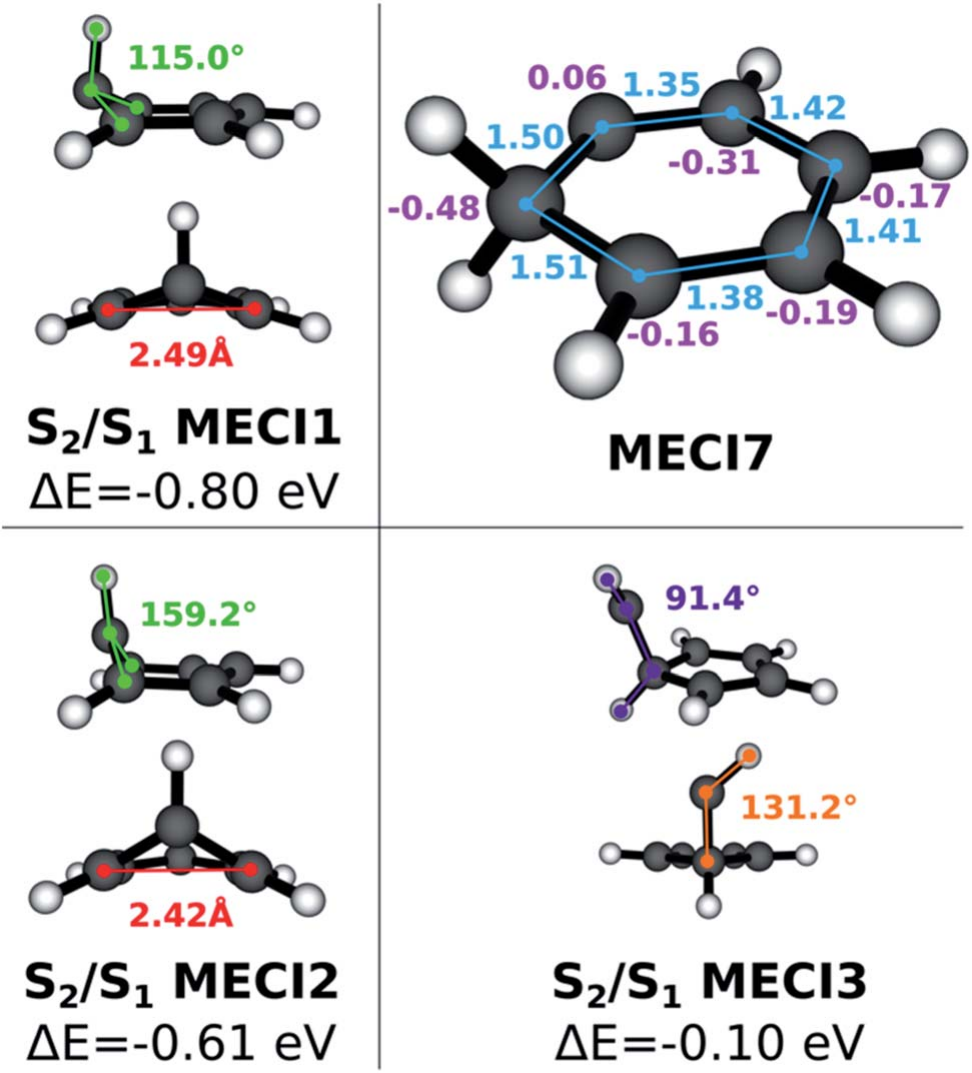
On the left and bottom right: S_1_/S_2_ MECI geometric structures; the pyramidalization dihedral value of the out-of-plane carbon atom is indicated in green; the distance between the bridge carbon atoms is indicated in red, in Angstroms; other interesting dihedrals and angles are pictured in violet and orange. The reported energy gaps are calculated with respect to the Franck Condon point S_2_ excitation energy. Top right: S_0_/S_1_ MECI7 geometric structure; carbon–carbon distances are indicated in light blue, carbon atoms Mulliken charges (FOMO-CASCI(6,5)/6-31G*) in purple.

Since benzene forms interesting photoproducts when excited to S_2_, we also explored the S_1_/S_2_ intersection seam in the Seam Dynamics step. Three S_1_/S_2_ MECIs were identified after the Seam Sampling and CI Selection (see Fig. 4). The energy of S_1_/S_2_ MECI1 is 0.8 eV lower than the FC point S_2_ excitation energy. The molecular geometry resembles S_0_/S_1_ MECI4, but the out-of-plane carbon atom protrudes much less prominently; the pyramidalization dihedral angle at this carbon atom is 115°. The geometry of the S_1_/S_2_ MECI2 resembles S_0_/S_1_ MECI4, but this MECI is ∼0.15 eV higher in energy than S_1_/S_2_ MECI1. Finally, S_1_/S_2_ MECI3 is very similar to S_0_/S_1_ MECI6, but the C_s_ symmetry is broken; the energy for this MECI is higher than the other two S_1_/S_2_ MECIs, although it still lies energetically below the S_2_ FC point. A comparison to previous literature is limited by the scarcity of research done on the benzene S_1_/S_2_ intersection seam. The S_1_/S_2_ MECI2 has been characterized previously,^64,67^ but to our knowledge the other two structures we found are undocumented. This provides further evidence that the NANR can be used as a discovery tool for unexplored excited state chemistry.

If geometry optimizations are started from the cone points sampled on S_1_ around each S_1_/S_2_ MECI, we observe two behaviors: either an S_1_ planar minimum energy point is reached, or one of the aforementioned S_0_/S_1_ MECIs are found. This is consistent with the moderate fluorescence reported in literature for benzene (*φ*_f_ = 0.053 at 254 nm).^69^ The moderate fluorescent quantum yield depends on the excitation wavelength, as the barrier separating the S_1_ minimum from the S_0_/S_1_ intersection seam can be overcome with sufficient vibrational energy (this is the explanation for the “channel three” phenomenon in benzene^70–72^). A minimum energy planar structure has been identified on the S_1_ potential energy surface; this geometry features average C–C bond lengths around 1.41 Å.^72,73^

As described in the previous section, when the exploration is started on states higher than S_1_, seam dynamics trajectories are started from the sampled cone points *in lieu* of geometry optimizations, in order to maximize the span of the sampled seam. More than 26 000 snapshots were collected in the Seam Sampling step and clustered following the procedure. Several known types of S_0_/S_1_ MECIs are reached during the CI Selection step. Additionally, another CI species (see Fig. 4), characterized by a carbene-like planar geometry that features a hydrogen transfer between adjacent carbon atoms and previously described by Lindner *et al.*,^29^ is found. The C–C bonds connected to the carbon atom carrying two hydrogen atoms have the length characteristic of a single bond; the adjacent C–C bonds have double bond character, while the last two have more of a hybrid character. The hydrogen donor carbon atom carries a slightly positive Mulliken charge, while the neighboring carbon atoms feature larger negative charges. Interestingly, this last MECI, labeled MECI7, is 1.5 eV lower in energy compared to MECI1 with FOMO-CASCI (perhaps due to the conserved planarity). This finding also contradicts the stereotypical picture of conical intersections in benzene featuring ring puckering. Other planar MECIs have been reported,^65^ but their energy is much higher (18.9 eV at the CASSCF (6,6)/cc-pvdz level of theory) than that of MECI1.

Without prior knowledge of possible CI types for benzene, our protocol was able to not only yield MECIs known in literature,^29,63–65^ but also to discover new potentially energetically accessible types, such as MECI3, MECI5, S_1_/S_2_ MECI1 and S_1_/S_2_ MECI3.

### Cone sampling and photoproduct search

In this step we focus on discovering what photoproducts are accessible from a given CI. From the literature, we know that when benzene is excited to S_1_, the minor photoproducts encountered are benzvalene and fulvene (see Fig. 2); some known key intermediates are prefulvene, a highly reactive biradical transient species found on the path thermally connecting benzene and benzvalene,^74^ and a carbene (1,3-cyclopentadienylcarbene) intermediate that serves as a precursor for fulvene. Both these intermediates are rather flexible structures: the energy barriers for the inversion at the radical center on the three-member ring of prefulvene and the rotation around the exocyclic single bond in the carbene intermediate have been computationally found to be negligible.^75^

When the molecule is excited to S_2_, Dewar benzene (see Fig. 2), fulvene and benzvalene are retrieved in a ratio of 1 : 2 : 5;^61^ with the estimated quantum yield for Dewar benzene being 0.006.^76^ Electrocyclic ring opening of Dewar benzene (yielding benzene) can be achieved *via* a conrotatory or disrotatory path. It has been demonstrated computationally that the conrotatory path is favored over the disrotatory path, which involves the formation of an extraordinarily strained *trans*-benzene (*cis*,*cis*,*trans*-cyclohexa-1,3,5-triene) intermediate.^77^

It must be noted, though, that benzene is always the major photoproduct of photoexcitation of benzene; while the quantum yields of Dewar benzene, benzvalene and fulvene are wavelength-dependent, these photoproducts are found in only trace amounts. The most abundant among them is benzvalene, with an experimental quantum yield which remains below 0.037 at best.^78^ The formation of these minor photoproducts falls within the chemical definition of rare events, which implies that many trajectories or purposefully biased initial conditions would likely be required to observe them with NA-MD.

**Fig. 5:**
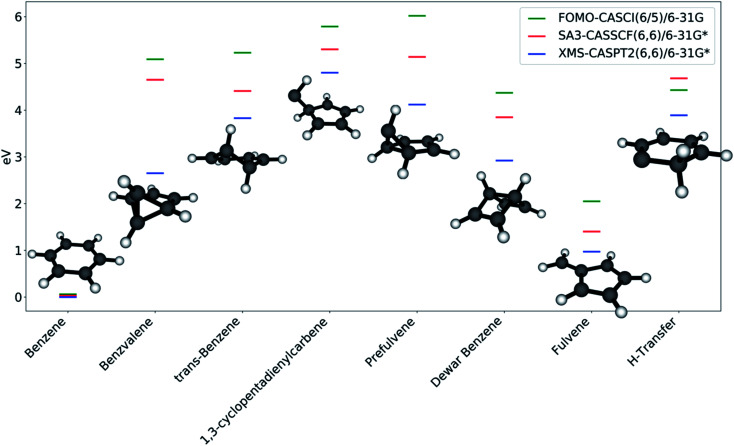
Stationary points identified on S_0_ by the NANR; fulvene and Dewar benzene were not discovered by the nanoreactor, and are added for comparison. All energies are referenced to the S_0_ equilibrium geometry of benzene at their respective level of theory.

The photoproducts identified by the collective search are shown in Fig. 5; Dewar benzene and fulvene, which were not found in this work, have been added for completeness. The reason for this is found in the nature of the intermediates that lead to a given photoproduct, as discussed in the next section: since *trans*-benzene and 1,3-cyclopentadienylpentene represent relatively deep minima on the S_0_ PES, the optimizations are not capable of overcoming the surrounding barriers to reach fulvene and Dewar benzene. In contrast, benzvalene can be retrieved in our framework due to the shallow nature of prefulvene as a minimum on the ground state. Most likely, adding dynamics effects to our protocol through short *ab initio* molecular dynamics on the ground state would improve the description of benzene photochemistry.

Benzene is, as expected, the stationary point reached most often in the S_0_ optimizations. Its energy is considerably lower compared to the energy of the other stationary points, due to the aromaticity of the molecule. All of the other photoproducts are at least 4 eV higher in energy (according to FOMO-CASCI) than benzene, with the exception of fulvene, which is closer (∼2 eV higher than benzene). The introduction of a better treatment of static (with CASSCF) and dynamic (with XMS-CASPT2) correlation lowers the relative energy of all the photoproducts, with shifts that are generally consistent from one molecule to the other. This is a further confirmation of the quality of FOMO-CASCI as a level of theory to describe the photochemistry of benzene, at least in a qualitative manner.

While the large majority of the photoproducts found have been discussed and characterized (either experimentally or theoretically) previously,^57^ MECI7 leads to a ground state minimum energy structure that is, to our knowledge, undocumented. The geometry, labeled in this work as “H-transfer” (see Fig. 5), is a planar carbene species featuring a hydrogen transfer between adjacent carbon atoms. Minor differences in the carbon–carbon bond lengths and in the Mulliken charges on the carbon atoms differentiate this stationary point from MECI7. The molecule is remarkably low in energy compared to the other photoproducts. This is likely due to the partial conjugation that is allowed to extend over a large section of the ring. It is important to note that this stationary point can only be reached from MECI7, from which this appears to be the only possible photoproduct, and not from the other discovered S_0_/S_1_ MECIs. Moreover, MECI7 was accessed when the exploration was started from S_2_; this may imply that the MECI resides in a more remote region of the S_0_/S_1_ intersection seam and requires specific conditions to be reached. It should also be kept in mind that the photoproduct search is performed through S_0_ optimizations, and that using dynamics could yield different results. This is why calculating paths connecting photoproducts is crucial - it provides a qualitative picture of how easily stationary points could interconvert.

**Fig. 6:**
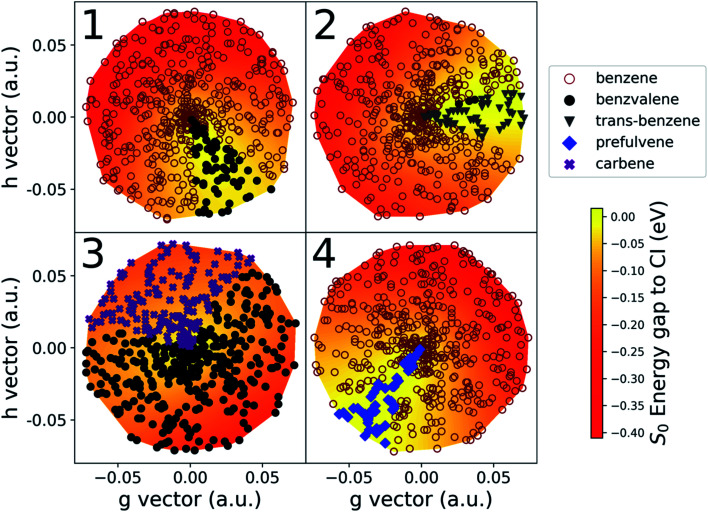
A graphic representation of the photoproduct distribution at MECIs 1 to 4. Each panel shows the lower portion of the CI cone, obtained through a triangulation performed on the 500 sampled cone points and color-mapped according to the energy difference with respect to the CI. The axes span the displacement along the *g* and *h* vectors. The symbols used for each of the cone points is chosen according to what photoproduct is reached from it upon S_0_ optimization (see upper right legend).

The details of the cone sampling and photoproduct search for MECI1, MECI2, MECI3 and MECI4 are shown in Fig. 6. MECI5, MECI6 and MECI7 are not represented because each of their sampled cone points leads exclusively to one photoproduct upon ground state geometry optimization (respectively, prefulvene, 1,3-cyclopentadienylpentene and H-Transfer). In MECI1, MECI2 and MECI4, the vast majority of the cone points yield benzene, with a total percentage of, 88.6%, 94.6% and 94.4%, respectively. MECI3 constitutes an exception, as optimizations started from cone points sampled on its cone do not lead to benzene, but only to benzvalene and the carbene intermediate, with a ratio of roughly 2 : 1.

Interestingly, though, the most abundant minor photoproduct retrieved (often the only one) differs in the three MECIs that feature benzene as the primary photoproduct. In MECI1, which features a symmetric structure, we observe the formation of benzvalene in 11.4% of the cases. In MECI2, where the symmetry is broken, the highly strained *trans*-benzene is observed in 5.1% of the total. Finally, MECI4, which retains the C_s_ symmetry but has a different orientation for the out-of-plane hydrogen, leads to prefulvene 5.6% of the time.

These results reveal that small geometric changes may substantially affect the photoproduct distribution from CIs. This raises the question of the probability of accessing any of these MECIs (or the region around them) after photoexcitation. For excitation to S_1_, the calculation of minimum energy paths on S_1_ starting from the FC point could provide some indication of accessibility (by quantifying any existing barriers). However, the question is more difficult when considering excitation to S_2_. In this case, the kinetic energy of the molecule after the transition to S_1_ should also be considered and we will address this in future work.

Another aspect worth noting in Fig. 6 is the fact that all cone points leading to a given photoproduct are grouped together in a sector on the CI cone; this suggests that there is indeed a well-defined volume of space around a CI leading to a given photoproduct that could be accessed depending on the velocity of a molecule transferring population through (or close to) the CI. We want to point out, though, that the volume of this space cannot be assumed to be simply equal to the percentage of cone points that lead to a photoproduct in our implementation (*e.g.* 11.4% for benzvalene at MECI1). This is because we are currently using geometry optimizations that follow the gradient. Introducing dynamic effects by sampling velocities at the cone points would likely change the ratio between the resulting photoproducts (possibly dramatically). This topic will be explored in future work. Indeed, it should also be noted that, typically, the minor photoproducts are reached through optimization of cone points sampled on the “flatter” portion (yellow parts in Fig. 6) of the cone, where the gradient is lower. Moreover, we know from literature that some of the stationary points we found are merely (rather unstable) intermediates in the formation of stable photoproducts. In other words, using dynamics instead of optimizations could possibly change the ratio in favor of benzene, or introduce new photoproducts in the cases of MECI2, MECI3 and MECI4, due to the amount of kinetic energy the molecule would have after decaying through a peaked CI. This would be sufficient to easily overcome low and medium barriers. Since, at this stage, the NANR does not offer information on the velocity distribution at a CI, nor a detailed description of the excited state PES topography, it is not yet possible to make numerical predictions about photoproduct ratios. However, the insight provided by these results could be used in the future to define new sampling schemes in the Cone Sampling step (*e.g.* selecting equally spaced points on a circle on the branching plane), in order to obtain a similar accuracy with less computational effort.

Further investigation with sampled initial velocities and comparison to nonadiabatic dynamics simulations results are needed in order to fully address the question about the dynamic effect on photoproduct distribution. Nevertheless, the NANR was able to identify a large portion of the known ground state stationary points for benzene, and discover a new one. The retrieval of these intermediates is indeed a good indication that our protocol is capable of identifying rare events, due to the systematic nature of the Cone Sampling step.

### Refinement

Many studies recognize the transient nature of *trans*-benzene, prefulvene and 1,3-cyclopentadienylcarbene.^74,75^ The newly found H-transfer species is also likely to be unstable. In order to verify our hypothesis, and to estimate how easily a photoproduct is formed, we calculate ground state minimum energy paths connecting the stationary points on the PES in the Refinement step.

Table 1 summarizes the GSM results for all pairs of photoproducts. Our calculated low barriers for prefulvene and the carbene species confirm that these are indeed transient structures serving as intermediates in the formation of other photoproducts, as shown in Fig. 2. Similarly, the calculated disrotatory ring opening path from Dewar benzene to benzene contains *trans*-benzene, as also found by Havenith *et al.*^77^

**Table 1:** S_0_ barriers (kcal mol^−1^) calculated with pyGSM (FOMO-CASCI(6/5)/6-31G) using a minimum number of 20 images between all possible pairs of S_0_ stationary points. When a number is not given, it indicates that the path connecting two species contains a notable intermediate

FROM	TO							
	Benzene	Benzvalene	Carbene	Prefulvene	*trans*-Benzene	H-Transfer	Fulvene	Dewar Benzene
Benzene	—	Prefulvene	Prefulvene	154.8	122.3	114.7	H-Transfer	*trans*-Benzene
Benzvalene	Prefulvene	—	16.3	18.7	Benzene	Benzene	Carbene	46.4
Carbene	Prefulvene	1.7	—	7.0	Benzene	Benzene	14.9	24.2
Prefulvene	17.7	1.4	4.3	—	16.5	Carbene	Carbene	13.3
*trans*-Benzene	1.2	Benzene	Benzene	32.7	—	Benzene	H-Transfer	19.7
H-Transfer	12.5	Benzene	Benzene	Carbene	Benzene	—	29.8	Benzene
Fulvene	H-Transfer	Carbene	102.2	Carbene	H-Transfer	84.9	—	93.9
Dewar benzene	*trans*-Benzene	65.6	58.0	49.8	40.1	Benzene	40.3	—

The ground state barriers separating benzene from all the other photoproducts are, as expected, extraordinarily high, ranging from 114 to 154 kcal mol^−1^; fulvene is also divided from the other photoproducts by similarly high barriers. Conversely, the transient species feature very low barriers, which are easily traversable. Some notable examples are the barriers between prefulvene and the carbene species, and the barrier between these isomers and benzvalene. Another noteworthy example of an extremely low barrier path is that from *trans*-benzene to benzene.

The newly discovered H-transfer species is also found to be a possible intermediate in the formation of fulvene from benzene or *trans*-benzene. Since the calculated path connecting the carbene intermediate to this H-transfer structure passes through benzene, one might speculate that two distinct possible pathways to photochemically form fulvene might exist, one including the prefulvene-carbene sequence of intermediates, the other featuring the H-transfer intermediate. However, the first path might be favored, since the cumulative prefulvene-to-carbene-to-fulvene barrier is ∼20 kcal mol^−1^, while the H-transfer-to-fulvene barrier is ∼30 kcal mol^−1^. It should be noted that the H-transfer-to-benzene barrier is lower (∼12 kcal mol^−1^), implying that benzene could be more easily formed than fulvene from this intermediate. Contrary to this, the prefulvene-to-benzene barrier has a comparable height to the overall barrier for the prefulvene-to-carbene-to-fulvene path, which could indicate a competition between benzene and fulvene in the photoproduct formation from prefulvene.

Finally, as stated previously, one might wonder about the accessibility of the S_0_/S_1_ MECIs. A qualitative picture can be obtained by calculating minimum energy paths connecting MECIs in the intersection space. A sizable barrier separating two MECIs in the seam space could indicate that the two CI types are only accessible from the FC point by following two distinct reaction channels. Conversely, low or non-existent barriers might suggest that the two CI types are similarly accessible from the same reaction channel, *i.e.* following a similar geometrical and electronic evolution.

**Fig. 7:**
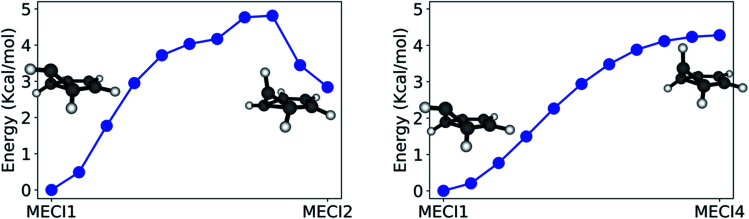
Paths constrained to the S_0_/S_1_ intersection space connecting MECI1 with MECI2 (left panel), and MECI1 with MECI4 (right panel). The paths are calculated using GSM (FOMO-CASCI(6/5)/6-31G) with a minimum number of 10 images. The *x* axis spans the Euclidean distance between images. The energies are given in kcal mol^−1^ and use the MECI1 energy as a reference.

The paths calculated during the last step of our workflow (Refinement) and connecting MECI1 with MECI2 and MECI1 with MECI4 are shown in Fig. 7 (see Fig. S2 in the ESI† for other paths), as these paths show the effect of breaking the CI symmetry and inverting the pyramidalization of the out-of-plane carbon atom.

The path calculated between MECI1 and MECI2 exhibits a ∼5 kcal mol^−1^ energy barrier, indicating that symmetry breaking is disfavored. On the other hand, the path between MECI1 and MECI4 is barrierless: this is due to the high conformational flexibility of the out-of-plane carbon atom. It should also be noted that, from these path calculations, MECI4 seems to be a very shallow minimum on the seam space.

Due to the absence of a proper energy barrier, we can imagine that the whole intersection seam region between MECI1 and MECI4 can be similarly accessed from the FC region. This is in agreement with experimental data, indicating benzvalene and fulvene, which have the prefulvene intermediate in common, as the most abundant minor photoproducts to be produced upon excitation to S_1_.^61^

The whole path connecting MECI1 and MECI2 lies below the S_1_ excitation energy at the FC point, and is therefore energetically accessible (supposing, of course, that no substantial barriers exist between the FC point and these MECIs). However, the small barrier calculated between MECI1 and MECI2 suggests that the two MECI types could be reached through slightly different reaction channels. In accordance with the literature, MECI2 could be principally accessed through excitation to S_2_; this would explain the formation of Dewar benzene observed only with an incident light of 203 nm, as MECI2 has *trans*-benzene in its photoproduct distribution and *trans*-benzene can rearrange to form Dewar benzene.^77^

The seam-constrained path connecting MECI7 with MECI1 (see Fig. S3†) exhibits a higher energy barrier (∼16 kcal mol^−1^). This suggests that the reaction channel needed to access MECI1 and MECI7 from the Franck Condon point might be quite different. Similarly, other paths presented in Fig. S2 in the ESI† might indicate that several reaction pathways are present on S_1_ for benzene. Further path calculations on the excited state could shed further light on this.

**Fig. 8:**
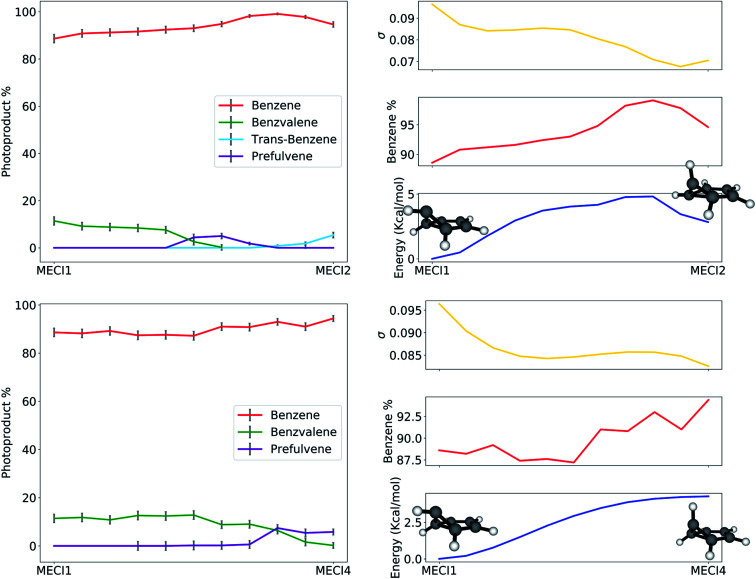
On the left: photoproduct distribution (as percentages) along the seam-constrained path between MECI1 and MECI2 (top) and MECI1 and MECI4 (bottom). The error bars are calculated with bootstrapping. On the right: a comparison between benzene retrieval percentage (in red), the slopeness *σ* (in yellow) and energy in kcal mol^−1^ with respect to MECI1 (in blue) along the MECI1–MECI2 and MECI1–MECI4 seam paths.

The path analysis can be taken a step further by using the path nodes to repeat the cone sampling and photoproduct search steps, in order to monitor how the photoproduct distribution changes along the path. The results for the MECI1–MECI2 and MECI1–MECI4 paths are shown on the left panels of Fig. 8. The photoproduct distribution changes quite smoothly along both paths. We observe a gradual decrease of benzvalene and an increase of *trans*-benzene going from MECI1 to MECI2, with a small amount of prefulvene being retrieved in the central part of the path. Looking at the topography of the CIs along the path (*σ* graphic, right panels of Fig. 8), we can observe a correlation between the slopeness, the energy and the benzene photoproduct ratio. While climbing up in energy along the path, the topography of the CIs becomes slightly less sloped, and at the same time the proportion of benzene photoproduct increases. This might suggest that a peaked topography offers a higher selectivity towards one type of photoproduct (benzene in our case), while a sloped one is less discriminatory. The inclusion of dynamical effects in future studies will clarify this point.

Similar conclusions can be drawn for the MECI1-MECI4 path (bottom panels in Fig. 8). The amount of benzvalene decreases while receding from MECI1, while that of prefulvene increases. A small increase in the benzene amount along the path corresponds to higher energies. Once again, the slopeness plot shows that a more peaked topography corresponds to higher energy CIs for this case.

Overall, the Refinement phase of the NANR deals with the current lack of dynamic effects we propose in the workflow by using path refinement techniques to gather qualitative information. This allows the user to acquire a holistic understanding of how the stationary points (both on the ground state and in the seam space) are connected, and how a given photoproduct can eventually be formed from the initial excitation of benzene.

## Conclusions

Predicting the outcome of a photochemical reaction without any prior knowledge is not an easy task, and to this day very few have tried to follow the evolution of a molecule from the photon absorption to the photoproduct with methods other than NA-MD. This is because of the intrinsic complexity of intersection hypersurfaces, which makes even the simple visualization of the problem challenging. Additionally, given that photochemical processes are intrinsically far from equilibrium, the static picture of stationary points, barriers separating them and the topography of the intersection space between two hypersurfaces does not provide enough information to allow accurate numerical predictions.

In this work, we show that the NANR can automate the exploration of excited state systems, predicting which photoproducts are accessible. The qualitative and, to some extent, quantitative data obtained provide important knowledge which can also be used to judiciously choose initial conditions for NA-MD in order to increase the likelihood of observing rare photochemical events.

The NANR was able to scan the intersection seam between different pairs of electronic states and report the structures of the MECIs encountered, most of which are separated by a significant energy barrier in the seam space. The cone sampling suggests that well defined volumes of space around the CI are dedicated to the formation of a given photoproduct. However, it is clear that a given photoproduct is reached only if specific conditions, including the velocity of the approaching molecule and the CI topography, are met. The information about the velocity distribution, usually obtained through nonadiabatic dynamics, deserves further attention, as it can help quantify the amounts of photoproduct formed through a CI.

The discovered S_0_/S_1_ MECIs could all be connected through seam-constrained paths, in agreement with previous conjectures about the universality of intersection connectivity.^79,80^ The photoproduct distribution and the topography of the CIs have been monitored along these paths, indicating a connection between the slopeness and the photoproduct selectivity: for the benzene molecule, lower energy CIs are more sloped and show less selectivity compared to the more peaked CIs found at higher energies in the seam space. This provides new insight into the connections between intersection properties and photochemical outcomes.

The identified photoproducts and ground state barriers between them are consistent with the information available in the literature:^56,70,71,73^ low barriers have been observed around transient species such as *trans*-benzene, prefulvene and carbene intermediates. Moreover, *trans*-benzene, a precursor for Dewar benzene, is easily reached when the photochemical exploration is started from S_2_, in agreement with experimental data that report traces of Dewar benzene only when using a 203 nm light source for the excitation. Furthermore, a new stationary point, featuring a planar structure and a H-transfer character, has been identified and proposed as a possible alternative intermediate in the formation of fulvene.

The NANR in its current state represents a first step toward acquiring the capability to simply plug a molecule (or several molecules) into a virtual reaction chamber, excite the system and get answers about its fate within hours without the need for a pre-existing knowledge of its photochemistry. Such tools can prove to be extremely valuable in accompanying and guiding experimental synthesis, where the role of light as a source of chemical energy is becoming increasingly important. This allows for a number of applications, including testing the effect of substituents on the same substrate, studying strategies to maximize the yield of a desired photoproduct, providing support for retrosynthesis planning and discovering unexpected photoreactions.

## Author contributions

E. P. contributed to the conceptualization, data curation, formal analysis, investigation, methodology, project administration, software, visualization and writing (original draft) of the presented work. D. L., A. M. C. and C. R. A. contributed to the software, data curation and formal analysis. K. C. T. contributed to the conceptualization, data curation, investigation, methodology and project administration. T. J .M. contributed to the conceptualization, funding acquisition, methodology, project administration, resources and supervision. All the authors equally contributed to the review and editing phases of the draft.

## Conflicts of interest

The authors declare no conflict of interest.

## Supplementary Material

SC-012-D1SC00775K-s001
