# Supplementary material for: The non-adiabatic nanoreactor: towards the automated discovery of photochemistry
Source: Chem Sci. 2021 Apr 29;12(21):7294–307. doi: 10.1039/d1sc00775k (PMC8171323; doi:10.1039/d1sc00775k)
Supplement: SC-012-D1SC00775K-s001 [file SC-012-D1SC00775K-s001.pdf]

Electronic Supplementary Material (ESI) for Chemical Science.
This journal is © The Royal Society of Chemistry 2021

SUPPORTING INFORMATION

The Non-Adiabatic Nanoreactor: Towards the Automated Discovery of Photochemistry

Elisa Pieri,[1,2] Dean Lahana,[1,2] Alexander M. Chang,[1,2] Cody R. Aldaz,[1,2] Keiran C. Thompson,[1,2] and Todd J. Martínez[1,2,*]

[1]Department of Chemistry and The PULSE Institute, Stanford University, Stanford, CA 94305

[2]SLAC National Accelerator Laboratory, 2575 Sand Hill Road, Menlo Park, CA 94025

Supplementary Information Table of Contents:

I. Detailed levels of theory and parameters for all calculation types
II. Seam meta-dynamics
III. Seam constraint
IV. Calculated seam-constrained paths
V. XYZ coordinates of MECIs
VI. XYZ coordinates of photoproducts

I. Detailed levels of theory and parameters for all calculation types

All the electronic structure calculations used in this study were performed using TeraChem[1-3]; the calculations described in this section were done in gas phase using the FOMO-CASCI(6,5)/6-31G.[4] Three singlet states have been included in the calculations and the FON temperature (kT) was set to 0.15 a.u.

a. Seam Meta-Dynamics

All seam trajectories, independent of the degenerate electronic states investigated, were run at 300K. The Bussi-Parrinello thermostat, combined with the velocity Verlet integrator, was used. The friction coefficient γ factor was set to 0.001 fs^{-1}. The integration time step is 1 fs. The constraint force was set to 10 $Hartrees^{-1}$ and the energy gap threshold between the two states to switch between a linear to a harmonic constraint is 0.005 Hartrees. Gaussian functions with a height of 0.002 Hartrees and a width of 1.0 Hartree were added each 25 fs, until a total meta-energy of 0.1 Hartrees was reached, at which point the calculation was stopped. This meta-energy was created by recording the cartesian space position vector of the molecule every 25 fs as a reference structure; then, the root-mean-squared distance (RMSD) between the molecule at a given timestep and each reference structure is used as the collective variable for the meta-dynamics. A quaternion algorithm[5] was used to properly align structures to minimize RMSD between two sets of vectors.

b. MECI search

The algorithm used for the MECI search is the Lagrange-Newton method.[6] The geometric optimization is carried out using geomeTRIC.[7]

c. Photoproduct search

The geometry optimizations have been performed using geomeTRIC.[7]

d. GSM paths

The GSM paths are calculated with either 20 images (on the ground state) or 11 images (for the S_1/S_0 seam constrained paths). For the ground state paths, geodesic interpolation[8] is used to construct the initial guess. In seam paths, the seam saddle points were obtained by using the climbing image algorithm.

II. Seam meta-dynamics

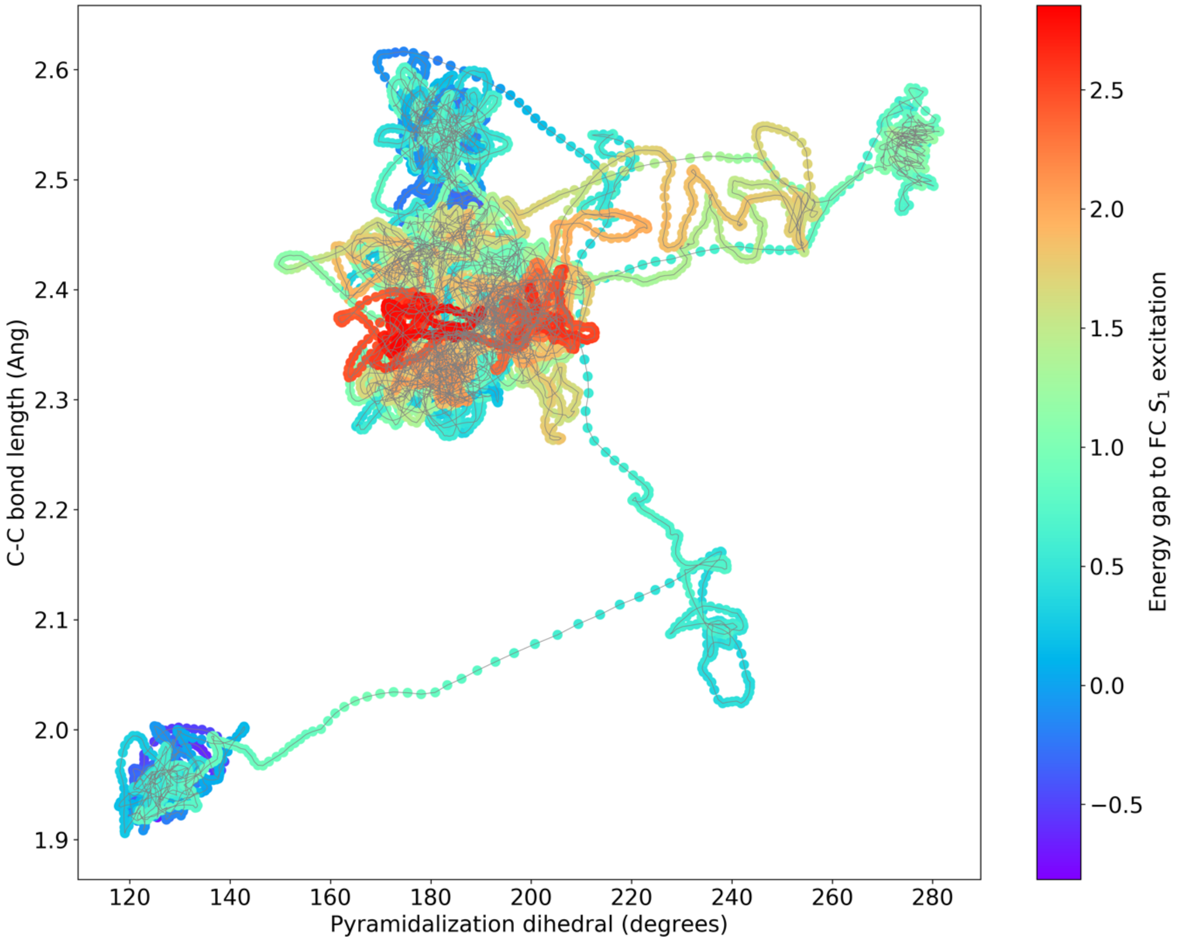

Figure S1. A color-mapped plot of the S_1/S_0 intersection seam. The monitored bond length and pyramidalization dihedral are the same as shown in Fig. 3 in the main text. From the FC point, the molecule explores many different wells in the seam space, as indicated by the clusters of points.

III. Seam constraint

The implemented seam constraint follows this equation for the added potential V_{con}:

$$V_{con} = \frac{1}{2}\, a\, \Delta E^2 \qquad if\ \Delta E \leq \theta$$

$$V_{con} = a\, \theta\, \Delta E - \frac{1}{2}\, a\, \theta^2 \qquad if\ \Delta E > \theta$$

ΔE is the energy gap between the electronic states. The values chosen for a and θ in all our seam dynamics are, respectively, 10 Hartrees^{-1} and 0.005 Hartrees.

IV. Calculated seam-constrained paths

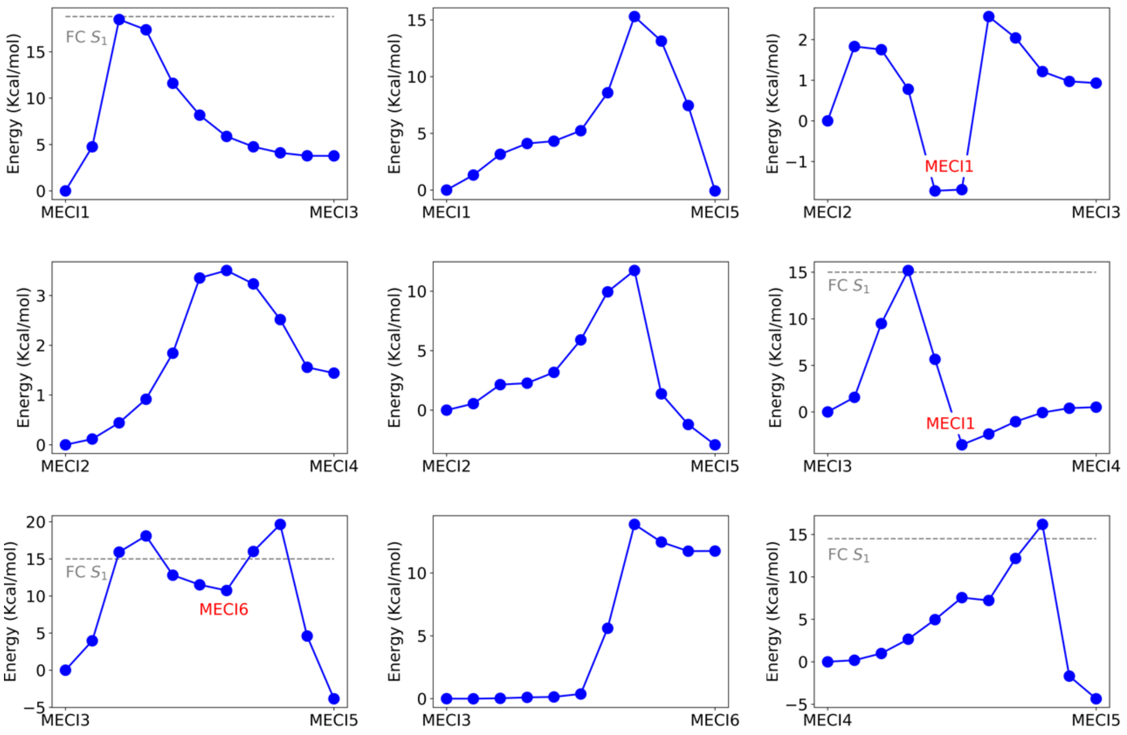

Figure S2. Seam-constrained paths between other pairs of MECIs, calculated with the GSM method at the FOMO-CASCI(6,5)/6-31G level of theory. When the path crosses the well corresponding to another MECI, it is indicated in red. When parts of the calculated path lie above the FC point, the FC energy level is indicated in grey. Many paths involving MECI6 could not be calculated due to discontinuities in the wavefunction.

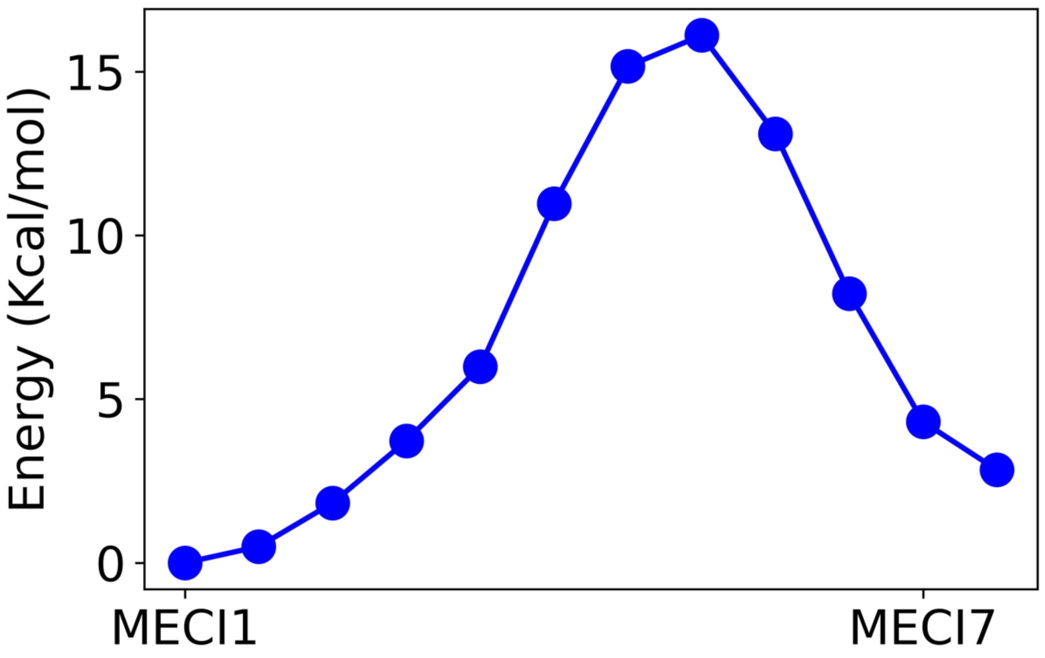

Figure S3. Seam-constrained path between MECI1 and MECI7, calculated with the GSM method at the FOMO-CASCI(6,5)/6-31G level of theory.

V. XYZ coordinates of MECIs

The coordinates are presented in the cartesian XYZ format.

a. S_1/S_0 MECI1

C	0.0688847386	-0.8663322174	0.8405303775
C	0.2842115509	-1.2236066766	-0.5649384879
C	0.2086544708	-0.1700165779	-1.4760246980
C	-0.2156864385	1.0248940469	-0.8945785179
C	-0.3484976623	1.0103752970	0.5653395706
C	0.6427331467	0.3477723934	1.3965536064
H	0.6268304569	0.5004864491	2.4612064907
H	-0.4559439609	-1.5534766508	1.4831471336
H	0.4297774225	-2.2483075810	-0.8514789271
H	0.4006543584	-0.2814026924	-2.5267146347
H	-0.4917475222	1.8958376297	-1.4589647067
H	-1.1498488253	1.5637475037	1.0259636711

b. S_1/S_0 MECI2

C	-0.3580947180	-1.2729349714	0.6612095018
C	0.5997579493	-1.1354847427	-0.4088125125
C	0.0155258636	-0.2145937203	-1.4011965651
C	-0.1429650522	1.1209184564	-0.9500108541

C	-0.0717531336	1.2503951665	0.4265469271
C	-0.1207234900	0.0315007996	1.2151446612
H	0.1166814701	0.1006182321	2.2654038646
H	-0.4734285629	-2.1360476407	1.2900331217
H	1.6665498806	-1.2446751965	-0.2835739882
H	-0.6370893278	-0.6164686522	-2.1582854645
H	-0.4822701632	1.9182625799	-1.5870131746
H	-0.1121886128	2.1984994792	0.9305478181

c. S_1/S_0 MECI3

C	-0.1977110000	-0.6960560000	0.7393890000
C	0.2204620000	-1.2138600000	-0.5468410000
C	0.1691360000	-0.1796170000	-1.5034400000
C	-0.2738950000	0.9935830000	-0.8813880000
C	-0.4902410000	0.7302110000	0.5374360000
C	0.8712800000	0.4059920000	1.2930900000
H	0.8610580000	0.5150680000	2.3647500000
H	-0.6520120000	-1.3168200000	1.4789600000
H	0.5712460000	-2.2072500000	-0.7205680000
H	0.5110870000	-0.2554510000	-2.5138000000
H	-0.3666900000	1.9517000000	-1.3432000000
H	-1.2240400000	1.2719400000	1.0946300000

d. S_1/S_0 MECI4

C	0.1958596814	-1.0670363391	0.8017975525
C	0.5243900463	-1.0758385831	-0.6199917984
C	-0.3223139358	-0.2765206670	-1.5159071055
C	0.0885928782	0.9958494646	-0.9056970583
C	-0.2807191985	1.2280794823	0.4873260889
C	-0.3297393163	0.1165034206	1.3375320369
H	-0.6724082644	0.1854814541	2.3532077011
H	0.4073324340	-1.9259178041	1.4125965980
H	1.4210573244	-1.5510834636	-0.9763730498
H	-1.3317012426	-0.5277480718	-1.7991458136
H	0.7505006758	1.6704862114	-1.4194057073
H	-0.4508227580	2.2277386764	0.8440957816

e. S_1/S_0 MECI5

C	0.2308828563	-1.0015139035	0.7632530545
C	-0.3230410542	-0.9195868770	-0.6046247148
C	0.6729311257	-0.2427939273	-1.5370563978
C	-0.6571779021	0.4999548129	-0.7930609871
C	-0.3264754525	1.1999494770	0.4153615945
C	0.2113769028	0.2746451156	1.3526034424
H	0.6350564680	0.5282029214	2.3008285626
H	0.6650161834	-1.8774220198	1.1942761288
H	-0.9342481943	-1.7019881374	-1.0033298240
H	1.6079950635	0.1254027475	-1.1513790387
H	-1.3617096472	0.8628799792	-1.5092515822
H	-0.4206063494	2.2522698114	0.5723798617

f. S_1/S_0 MECI6

C	0.4787526498	-0.8769215601	0.5445490146
C	-0.0883269514	-0.5479889865	-0.8315802260
C	0.8458711410	-0.7095380967	-1.9562897351
C	-0.6231453241	0.8641827419	-0.6227224122
C	-0.4313719503	1.2266626235	0.6544233468
C	0.2504030030	0.1508313700	1.3757356226
H	0.5184612587	0.1971944032	2.4112678348
H	0.9673159092	-1.8018204709	0.7668244919
H	-0.9399615978	-1.2053641230	-1.0084884119
H	1.4113528295	-0.9323809870	-2.8213100690
H	-1.0826424622	1.4337315271	-1.4024271463
H	-0.7233697298	2.1565469939	1.0975749993

g. S_1/S_0 MECI7

C	0.4905818555	1.3150803121	0.3123429855
C	1.6177399725	0.8895477460	-0.4455051027
C	1.6219805265	-0.3769312423	-1.0613540877
C	0.5483522597	-1.2321393988	-0.9488207022
C	-0.6848949863	-0.8667828814	-0.1673003956
C	-0.5771863922	0.4988079499	0.4403122552
H	-1.5659155484	-0.9030167461	-0.8024791239
H	0.4978297233	2.2815847345	0.7771121798
H	2.4660292219	1.5373696755	-0.5474694874
H	2.4808500976	-0.6792224254	-1.6288568578
H	0.5710115621	-2.1932663329	-1.4253815286
H	-0.8684938905	-1.5918639967	0.6209683081

h. S_2/S_1 MECI1

C	-0.9286120000	0.2747000000	-0.2148970000
C	0.1180550000	1.1883700000	-0.4841420000
C	1.2408000000	0.6811330000	-1.3059200000
C	1.6236400000	-0.7181170000	-1.0081000000
C	0.5650610000	-1.6165800000	-0.7348400000
C	-0.6869750000	-1.0952300000	-0.3825990000
H	-1.4627300000	-1.7836000000	-0.1062240000
H	-1.8567500000	0.5946740000	0.2169290000
H	0.0523311000	2.2081200000	-0.1555380000
H	0.8620600000	0.6659820000	-2.3370600000
H	2.6382500000	-1.0655200000	-1.0549600000
H	0.7260790000	-2.6755800000	-0.6811690000

i. S_2/S_1 MECI2

C	-0.1121944263	-1.0392643620	0.7635953408
C	0.5872833825	-1.2530828864	-0.4854377596
C	0.0897989674	-0.2507305821	-1.3324933587
C	-0.4818850500	0.9161915865	-0.7712465250
C	-0.2328995342	1.2005318228	0.5927630423
C	0.2651763903	0.1898977123	1.4286164577
H	0.7561349555	0.2910783301	2.3698232938

H	-1.0508349023	-1.5408648608	0.9838470406
H	1.2813543573	-2.0295836872	-0.7143175437
H	0.1724491576	-0.3490697261	-2.3975487922
H	-0.8847996717	1.6732208855	-1.4095610161
H	-0.3895836261	2.1916757673	0.9719598200

j. S_2/S_1 MECI3

C	0.3274196107	-0.9612418231	0.7244430988
C	-0.2721985026	-0.7802751019	-0.6658910453
C	0.9745839716	-0.7480087051	-1.4838221509
C	-0.6385660662	0.6759492817	-0.6204765260
C	-0.3925596101	1.2093585832	0.7176398298
C	0.1684837703	0.2612659341	1.4889484011
H	0.4922268557	0.3812921101	2.5008226353
H	0.8117505166	-1.8537870031	1.0563161746
H	-1.0506341268	-1.4657779453	-0.9531335031
H	1.2477469002	-0.1516497833	-2.3335606839
H	-1.0397383922	1.2242507474	-1.4461980518
H	-0.6285148272	2.2086238054	1.0149119213

VI. XYZ coordinates of photoproducts

The coordinates are presented in the cartesian XYZ format.

a. Benzene

C	0.2158396889	-1.0777190893	0.8544122982
C	0.2993681968	-1.2545806771	-0.5239473102
C	0.0832757917	-0.1769474180	-1.3783533808
C	-0.2159098949	1.0777247691	-0.8544428341
C	-0.2991029080	1.2546434669	0.5239691216
C	-0.0836148800	0.1768773600	1.3783365208
H	-0.1483507242	0.3130079294	2.4410344392
H	0.3825548780	-1.9084948627	1.5132351922
H	0.5304310234	-2.2217874882	-0.9278705522
H	0.1470896481	-0.3133973911	-2.4410723381
H	-0.3820190759	1.9086863515	-1.5131793235
H	-0.5295616053	2.2219873171	0.9278776135

b. Benzvalene

C	-0.1806342775	-0.7523613874	0.7627751428
C	0.1224766952	-1.3037798585	-0.6101984556
C	0.3106649726	-0.2238112123	-1.3930464139
C	0.1188479394	0.9658001424	-0.4824049520
C	-0.8779680342	0.5954470608	0.6101348257
C	0.5446780995	0.5794820377	0.9307119825
H	1.1584293230	1.0182716270	1.6831591807
H	-0.3756876119	-1.3842703887	1.6056842593
H	0.1704282069	-2.3401116759	-0.8675650487
H	0.5465518574	-0.1785259242	-2.4347042735
H	0.2101680847	1.9754182613	-0.8299539736
H	-1.7479631457	1.0484313302	1.0254106651

c. 1,3-cyclopentadienylcarbene

C	-0.0579148877	-0.5307055685	0.7849069100
C	0.3593305985	-1.1161822952	-0.5355471170
C	0.1516930403	-0.2146378430	-1.5208670939
C	-0.4276171973	0.9894267081	-0.9509297725
C	-0.5856508254	0.8491633795	0.3807714362
C	1.1015663246	0.0022189646	1.5716837155
H	0.7720967895	0.1543124636	2.6071866166
H	-0.7967473894	-1.1235406422	1.3097032930
H	0.8081275866	-2.0817983898	-0.6355178651
H	0.3982263985	-0.3353769631	-2.5551076278
H	-0.7039085365	1.8552125555	-1.5173998192
H	-1.0195246721	1.5513446689	1.0601442154

d. Prefulvene

C	0.3108635348	-0.7411129259	1.1373716824
C	0.8715893417	-0.9525860255	-0.1367764089
C	0.4294094119	0.1423977728	-1.0662661230
C	-1.0303721648	0.2163819538	-1.2774150598
C	-0.4739374332	1.0588170394	-0.2001468879
C	-0.4774512447	0.4228407273	1.1626008651
H	-0.9986281460	0.8154982959	2.0096401841
H	0.4486996514	-1.3953134589	1.9725953616
H	1.5207789954	-1.7550075218	-0.4160106081
H	1.0988196250	0.5638317083	-1.7844495570
H	-1.7775085164	-0.5453463003	-1.2480761100
H	-0.4332477848	2.1202263420	-0.3152206770

e. *trans*-Benzene

C	-0.0644944482	-1.1293836465	0.9348240205
C	0.5779409901	-1.2942704057	-0.4119749083
C	0.2906939617	-0.2142688665	-1.1781304210
C	-0.4290349587	0.9636617945	-0.6370113521
C	-0.5562382626	1.1777145906	0.6948046381
C	0.2441990062	0.1486589650	1.4394175506
H	1.2961629058	0.3878218831	1.4858042835
H	-1.1127075520	-1.3883729930	0.9353622261
H	1.0367887734	-2.1798006007	-0.7994024838
H	0.5360670503	-0.2034678002	-2.2233997462
H	-0.8073993853	1.6769183074	-1.3448167555
H	-1.0119787091	2.0547897494	1.1045228043

f. Dewar-Benzene

C	-0.8819000000	-1.0352800000	0.6028700000
C	0.4766920000	-0.8370510000	-0.0857615000
C	0.4499130000	-0.0698916000	-1.4161800000
C	0.4622990000	1.1476500000	-0.8261260000
C	0.4907040000	0.5782000000	0.6001270000
C	-0.8697650000	0.1821910000	1.1930600000

H	-1.5700100000	0.7168690000	1.7992200000
H	-1.5953600000	-1.8309700000	0.5641300000
H	1.2620000000	-1.5489500000	0.0919087000
H	0.3805230000	-0.3986790000	-2.4314400000
H	0.4063840000	2.1492700000	-1.1966000000
H	1.2859700000	0.8670560000	1.2628300000

g. Fulvene

C	-2.6026057203	1.3871448611	0.0000008362
C	-2.0998104646	-0.0041010188	0.0000341681
C	-0.3078950892	1.4299788809	-0.0000082262
C	0.9612311101	1.8886288979	-0.0000236431
H	-2.7226944404	-0.8777066313	0.0000583850
C	-1.5440850974	2.2340156299	-0.0000241958
H	1.8013905416	1.2178229939	-0.0000094385
H	-0.0794504378	-0.8200569467	0.0000476597
C	-0.7444478833	0.0214097374	0.0000288071
H	1.1784259961	2.9415667689	-0.0000507319
H	-3.6400746815	1.6606961918	-0.0000024313
H	-1.5706841572	3.3062003005	-0.0000511893

h. H-Transfer species

C	0.5023250990	1.2989627019	0.3043378192
C	1.5731129327	0.8820456552	-0.4297160249
C	1.6188522680	-0.4067746805	-1.0809884898
C	0.5611404550	-1.2425091299	-0.9644885583
C	-0.6377942705	-0.8336686826	-0.1706225385
C	-0.6863319613	0.5123229856	0.5099499348
H	-1.5286934815	-0.8802629673	-0.7943682471
H	0.5431878027	2.2717617305	0.7585836758
H	2.4270529812	1.5269987076	-0.5381762543
H	2.4904064491	-0.6763411823	-1.6446754971
H	0.5663921520	-2.2074466676	-1.4374028730
H	-0.8317660237	-1.5659210770	0.6111354975

References:

(1) Ufimtsev, I. S.; Martinez, T. J. Quantum Chemistry on Graphical Processing Units. 3. Analytical Energy Gradients, Geometry Optimization, and First Principles Molecular Dynamics. *J Chem Theory Comput* **2009**, *5*, 2619.
(2) Ufimtsev, I. S.; Martinez, T. J. Quantum Chemistry on Graphical Processing Units. 2. Direct Self-Consistent-Field Implementation. *J. Chem. Theory Comput.* **2009**, *5*, 1004.
(3) Ufimtsev, I. S.; Martinez, T. J. Quantum Chemistry on Graphical Processing Units. 1. Strategies for Two-Electron Integral Evaluation. *J. Chem. Theory Comput.* **2008**, *4*, 222.
(4) Slavíček, P.; Martínez, T. J. Ab initio floating occupation molecular orbital-complete active space configuration interaction: An efficient approximation to CASSCF. *J. Chem. Phys.* **2010**, *132*, 234102.
(5) Coutsias, E. A.; Seok, C.; Dill, K. A. Using quaternions to calculate RMSD. *J. Comp. Chem.* **2004**, *25*, 1849.

(6) Manaa, M. R.; Yarkony, D. R. On the intersection of two potential energy surfaces of the same symmetry. Systematic characterization using a Lagrange multiplier constrained procedure. *J. Chem. Phys.* **1993**, *99*, 5251.
(7) Wang, L.-P.; Song, C. Geometry optimization made simple with translation and rotation coordinates. *J. Chem. Phys.* **2016**, *144*, 214108.
(8) Zhu, X.; Thompson, K. C.; Martinez, T. J. Geodesic interpolation for reaction pathways. *J. Chem. Phys.* **2019**, *150*, 164103.
